# Experiences Applying Technology to Overcome Common Challenges in Pharmacy Practice-Based Research in the United States

**DOI:** 10.3390/pharmacy8020093

**Published:** 2020-05-30

**Authors:** Stephanie A Gernant, Omolola A. Adeoye-Olatunde, Matthew M. Murawski, Heather Jaynes, Betty Chewning, Lyndee M. Knox, Moises Martinez III, Jon C. Schommer, Margie E. Snyder

**Affiliations:** 1 Department of Pharmacy Practice, University of Connecticut School of Pharmacy, 69 North Eagleville Rd., Storrs, CT 06226, USA; 2 Department of Pharmacy Practice, Purdue University College of Pharmacy, 640 Eskenazi Avenue, Indianapolis, IN 46202, USA; adeoyeo@purdue.edu (O.A.A.-O.); hwroblew@iupui.edu (H.J.); snyderme@purdue.edu (M.E.S.); 3 Department of Pharmacy Practice, Purdue University College of Pharmacy, 575 Stadium Mall Drive, West Lafayette, IN 47907, USA; murawski@purdue.edu (M.M.M.); marti880@purdue.edu (M.M.III); 4 Social and Administrative Sciences Division, School of Pharmacy, University of Wisconsin-Madison, 777 Highland Ave., Madison, WI 53705, USA; betty.chewning@wisc.edu; 5 LA Net Community Health Resource Network Collaboratory, 800 East Ocean Blvd, Suite 104, Long Beach, CA 90802, USA; lyndee.knox@lanetpbrn.net; 6 College of Pharmacy, University of Minnesota, 308 Harvard St. SE, Minneapolis, MN 55455, USA; schom010@umn.edu

**Keywords:** information technology, evidence-based pharmacy practice, health services research, practice-based research

## Abstract

Despite the importance of pharmacy practice-based research in generating knowledge that results in better outcomes for patients, health systems and society alike, common challenges to PPBR persist. Herein, we authors describe PPBR challenges our research teams have encountered, and our experiences using technology-driven solutions to overcome such challenges. Notably, limited financial resources reduce the time available for clinicians and researchers to participate in study activities; therefore, resource allocation must be optimized. We authors have also encountered primary data collection challenges due to unique data needs and data access/ownership issues. Moreover, we have experienced a wide geographic dispersion of study practices and collaborating researchers; a lack of trained, on-site research personnel; and the identification and enrollment of participants meeting study eligibility criteria. To address these PPBR challenges, we authors have begun to turn to technology-driven solutions, as described here.

## 1. Introduction

Practice-based research occurs in settings where patients typically receive care, and groups of practices, providers and other healthcare entities often partner with each other and other researchers to develop practice-based research networks (PBRNs) [1]. Frequently affiliated with academic institutions, PBRNs draw on the insight of clinicians to identify and frame research questions. By linking these questions with rigorous research methods in this unique setting, PBRNs produce research findings relatively quickly and findings that are relevant and easily translated into everyday practice. Initially gaining popularity in the mid-1960s within primary care, PBRNs now exist across the world and focus on various settings, disease states, populations and professions, including pharmacy. As of February 2020, 186 PBRNs are registered with the United States Agency for Healthcare Research and Quality, in which six are pharmacist-based and another 72 conduct medication-related research [1]. Pharmacy practice-based research (PPBR) in the United States has potential for continued growth because Americans take more medications today than ever before, and pharmacists are the healthcare professionals who are most accessible and the highest trained on medications [2]. Despite PPBRs’ importance in generating knowledge resulting in better outcomes for patients, health systems and society alike, common challenges persist. Notably, inadequate financial support stunts PPBR by reducing the time and resources needed by clinicians and researchers to participate in study activities [3,4]. Moreover, PPBR scientists often encounter primary data collection challenges due to unique data needs and data access/ownership issues. Other well documented barriers to PPBR include (but are not limited to) the wide geographic dispersion of study practices and collaborating researchers; a lack of trained, on-site research personnel; and the identification and enrollment of participants meeting study eligibility criteria [3,4]. To address these challenges, pharmacy researchers have begun to turn to technology-driven solutions. Herein, we authors from various regions of the United States describe the PPBR challenges our research teams have commonly encountered, and our experiences using technology-driven solutions to overcome these challenges. Of important note, we authors assert our neutrality and neither endorse nor discourage the use of any particular single software program or IT product; the experiences below are our own and we have no investments, relationships, competing interests or other conflicts of interest—financial, intellectual or otherwise—with any technology discussed. Rather, we share our experiences using the technologies available through our respective institutions and would encourage readers to explore the comparable available technologies for their own use to address PPBR challenges.

## 2. Technology Used to Facilitate Pharmacy Practice-Based Research (PPBR)

A summary of the authors’ experiences with using an array of technology to overcome common PPBR challenges as framed via the Technology Acceptance Model (TAM) is provided in Table 1 and further described in the sections below [5]. Developed in the 1980s, the TAM has been widely referenced in the technology acceptance field and used in healthcare for over 20 years [6,7,8,9,10]. It consists of six constructs, namely external variables, perceived usefulness, perceived ease of use, attitude toward using, behavioral intention to use and actual system use. We applied select constructs of the TAM, “perceived usefulness” and “perceived ease of use”, to frame the authors’ experiences with technology. First, during a conference call, the authors discussed the challenges they encountered during studies in which one or more members collaborated. From this discussion, a shared Google-Doc file relating challenges to TAM constructs was developed and the researchers were prompted to individually expand on their experiences, as well as add any additional challenges/technological solutions they believed were pertinent. All authors could view and comment on each other’s contributions; summaries were developed by one author and shared with the group, at which point any discrepancies were resolved by discussion and consensus.

### 2.1. Smartphones, Tablets and other Mobile Devices

In our prior PPBR experiences, we authors found that mobile devices (e.g., smartphones, surfaces and tablets) are important to practice-based research because they are often fundamental to the use of other technology, including the technology-driven solutions described within this manuscript. Indeed, mobile devices have been used to facilitate an innumerable range of interventions, including everything from direct patient care and monitoring, to inter-professional communication and collaboration; however, mobile devices’ utility in PBRN research extends far beyond intervention delivery [11,12]. For example, we authors experienced that the identification, recruitment and enrollment of patient subjects is particularly challenging in community-based PPBR because community pharmacists’ services in the United States are rarely provided on an appointment basis. Rather, as the most accessible healthcare provider, U.S. community pharmacists more often provide care on a walk-in basis, leaving researchers unable to predict when potential patient subjects will present [13,14]. Moreover, we found PPBR challenging often due to the lack of resources required to physically position trained research personnel on-site at remotely located study locations. In response, not only have mobile devices and other handheld technologies shown promise in coordinating/training research personnel (as described in several sections below) but have also facilitated PPBR by their use in informed consent processes [15].

The authors used guidance for obtaining informed consent via electronic processes (i.e., electronic informed consent or ‘eIC’) issued by the U.S. Department of Health and Human Services (HHS) Office for Human Research Protections (OHRP) and the Food and Drug Administration (FDA), and as such, have utilized these practices to overcome the identification, recruitment and enrollment challenges. For example, handheld devices utilizing Research Electronic Data Capture (REDCap) e-form software (as further described below) allowed the authors to perform subject consent off-site and capture electronic signatures. For example, e-consent processes were used in Snyder et al.’s recent usability-study that video-recorded pharmacists as they prepared to deliver comprehensive medication review (CMR) services to patients. To do this, authors first obtained a ‘waiver of documented consent’ from the university’s Institutional Review Board (IRB) which allowed authors to speak with patients by phone prior to the patient’s CMR appointment. During this call, authors explained the study and obtained the patient’s verbal consent for authors to video-record their pharmacists as they prepared for the patient’s upcoming CMR appointment. Then, once the patient appeared at their community pharmacy for their CMR appointment, the patient watched a pre-loaded video on a study iPad that reviewed the consent and HIPAA authorization documents. After watching the video, the patient was navigated to REDCap, where pre-loaded consent documents allowed patients to ultimately provide authors with written consent [16]. This technology can also be useful for researchers seeking to enroll non-English speakers when no on-site translation services are available. Specifically, as part of the authors’ ongoing PPBR study, on-site research personnel use handheld devices to access e-forms/surveys (as described below) containing brief translated recruitment materials and scripts for making telephone contact/hand-offs to off-site translators [15].

Despite their helpfulness, we have encountered several limitations to the use of mobile devices in PPBR. First, mobile devices’ most recognizable benefit to PPBR—their portability—is limited because the devices are often expensive and fragile. Researchers must not only invest in the devices themselves, but also anti-theft and protection software and accessories. Their mobility is especially challenging because the more they are used, the more they risk breakage and loss. Similarly, their increased risk of loss compared to the traditional desktop makes mobile devices more likely to cause data breaches. Furthermore, devices must receive regular maintenance and care, like charging, software updates, and virus protection, as well as device replacement after a period of time; this results in ongoing costs. Lastly, the use mobile devices in PPBR is challenging because researchers must be knowledgeable and comply with a litany of regulation. Not only must the use of mobile devices comply with the researchers’ respective university IRBs, but also many federal and state regulations. The protection of private health information under HIPAA is the most notable regulation, but researchers interested in using or developing mobile technology for PPBR should also be aware of the regulations of the U.S. Federal Trade Commission (FTC). Despite these challenges, we authors plan for the continued use of mobile devices to facilitate PPBR because they are helpful not only with the identification and consent of patients, but also with data collection and research team communication/training, as described in the following sections.

### 2.2. Electronic Form and Survey Software

The authors used several electronic tools to develop and deploy novel electronic data collection forms (i.e., e-forms/surveys) [4,16,17,18,19,20,21,22,23]. In our experiences, we found that these ‘e-form/survey’ programs required little-to-no informatics training and were often accessible via desktop computers, tablets and other mobile devices alike. Basic features allow researchers to create items such as fill-in the blank, single–multiple choice and “select all that apply” questions, whereas more advanced features can apply skip-logic, auto-validate data, or calculate fields. E-form/survey programs are usually housed on a server, so no extra downloads are required and researchers’ data can be subsequently stored on that server. Server-stored data can then be downloaded in files compatible with the analysis programs such as Excel, SPSS Statistics or R-software. In addition, we found that e-form/survey programs often have off-line features that allow researchers to collect data without an internet connection.

While many different e-survey platforms exist, and we in no way endorse or discourage the use of any single product, we researchers have had the most experience with REDCap, the Research Electronic Data Capture tool [24]. Developed in 2004 by Vanderbilt University with support from the U.S. National Institutes of Health, REDCap l was created to facilitate the design of clinical and translational research databases [24]. Specifically, the developers aimed for REDCap to support small, investigator-initiated studies via a user-friendly interface and as such, the tool has been used in studies around the world [24]. Accordingly, we found that REDCap was specifically useful for PPBR because the development, testing, launching and utilization of data collection databases requires little-to-no IT/informatics specialists’ support. REDCap allowed us to import common data collection tools (i.e., validated surveys and instruments) as well as share novel data collection forms with other institutions. The software is distributed to institutional partners at no cost but is neither open source, nor permitted for commercial purposes; as such, we encountered instances where we were charged small fees to cover the cost of the internal IT support staffing. Another e-form/survey software program we commonly used was developed by a private corporation, Qualtrics LLC. Like REDCap, we found that the Qualtrics software allows researchers to disseminate e-forms/surveys via hyperlinks and can present simple reports (i.e., descriptive statistics) on entered data.

We found that other e-form/survey platforms were usually accessible to pharmacy researchers via their academic institutions at little-to-no cost. These e-form/survey platforms may also offer free, publicly available versions online via vendors like SurveyMonkey, or Qualtrics, or free versions via social-media companies like LinkedIn and Facebook. However, we found that even if a certain vendor’s e-form/survey software is accessible, free and/or used by other organizations/universities for their research programs, that our own institutions may not necessarily contract with these vendors as well. Assuring that a vendor’s program is contracted with our respective university is important because we found that free and contracted versions of software programs may have various features. For example, we found that while publicly available e-forms/surveys may offer HIPAA-compliant features and may state that they comply with international privacy laws like the European Union’s General Data Protection Regulation (GDPR), federal privacy regulation in the United States has not kept pace with the rate of technology’s advancement and accessibility [25]. As such, any researcher interested in using e-form/survey platforms should know that free and contracted versions may have different levels of protection. Furthermore, while publicly available e-forms/surveys are seemingly “free to use,” data access and ownership issues remain. Specifically, whereas publicly available e-form/survey programs may allow researchers to use their software without costs, we have encountered instances where data collected has been kept behind a paywall. Collectively, we authors found the greatest success when we utilized our university-contracted software rather than free-to-use versions and encourage other PPBR study teams to regularly check with their respective organizations/university’s IT department before choosing a product for their project.

We used several other ubiquitous software programs, like Microsoft Excel and Access, as they are commonly used by health-systems and clinicians alike to collect data [26]. However, we experienced that e-form development on Access and Excel requires a moderate level of skill, and data entered directly into Excel can be prone to data-entry errors, over-writing and erroneous deletion. Conversely, we have found that the REDCap and Qualtrics programs offer features like data validation, force-functions and data repositories that decrease the likelihood of such data-collection mistakes. Overall, we found that the use of e-form/survey programs facilitated PPBR in a wide variety of methods, subjects and settings and are particularly helpful to conduct surveys and collect data [15,16,23,27].

### 2.3. Video/Audio-Recording Devices and Software

Traditionally, we authors have relied on digital recorders, tape-recorders and our own hand-written notes to collect study participants’ words and actions. Today, we are beginning to use digital applications designed for desktops and mobile devices, like Dragon, Google Voice and InfraWare Dictation (InfraWare Inc.) to record video and audio data. Several of the authors have used HIPAA-compliant versions of these applications to record semi-structured interviews, focus-groups, contextual inquiries and investigator observations in past and current PPBR research projects [15,23,28,29,30]. While Dragon and Google Voice use artificial intelligence to transcribe recordings, the InfraWare records dictation via smart-phone, computer, or tablet for the researcher to upload to the application’s web-based platform for human transcription.

Our experiences using the InfraWare software for PPBR were mainly positive, but we are neither endorsing nor discouraging its use. As a cloud-based application, we found that we needed neither cellular data nor Wi-Fi to use InfraWare Dictation’s features because users can record audio data without internet and later upload data to the platform when connectivity is re-established, minimizing the risk of data breaches. Once uploaded, the InfraWare Dictation’s platform provides a secure venue for healthcare transcriptionists to transcribe audio files, which can be subsequently downloaded as a Word, RTF or PDF file. Upon logging into the application’s portal, all of our audio recordings, transcripts and progress on partially transcribed files are viewable. While we authors were able to download the InfraWare Dictation application for free (eliminating the need for researchers to purchase HIPAA-compliant recording devices), transcription services were associated with a cost, which we found could be prohibitive for some studies. We found that the application generally required no more skill than using hand-held audio devices, and we were supported by online training videos, user guides and a designated support contact for our respective university. However, we found that pausing and restarting recordings was complicated by mobile notifications. As such, we found we could avoid the risk of unintentionally stopping a recording in InfraWare Dictation by placing recording devices in airplane mode.

In addition to facilitating data collection and subject recruitment, we found that mobile audio/video devices, like GoPros, have shown promise in training research personnel, especially for personnel working at off-site study locations. GoPros, and other handheld recording devices, are usually small, wearable, light-weight audio/video cameras that are often purchased with proprietary video-editing software. We found that these hand-free devices allowed trainees to watch and listen to pre-recordings of trainers performing a wide range of research activities conducted in the first-person (i.e., protocol activities, data collection, recruitment processes and administrative duties). Similarly, videos can convey spatial and process information more quickly than written instructions and can be especially helpful for research projects that have multiple, temporary, or off-site personnel. For example, some of the authors used GoPro training videos to facilitate PharmD students’ integration in PPBR. Specifically, our ongoing research examines how PharmD students, in their final year of advanced pharmacy practice experiences (APPEs) could act as pharmacist-extenders in delivering transitions of care services over a period of two months [31,32]. In addition to assisting pharmacists in delivering the standard intervention, the students were also tasked with collecting and entering the study data in this practice-based project. Video recordings made on GoPros and other hand-held devices helped to quickly train students step-by-step on research protocols as they rotated in and out of the study and helped reduce variability in the research project’s functions.

However, while GoPro training videos can last as enduring education and can help facilitate the training of multiple research personnel that rotate through studies, we found that their usefulness diminishes in relation to how frequently a study’s protocol updates. Similarly, researchers must have both access to and working knowledge of video-editing software, as well as recording accessories like microphones, SD cards (for memory storage) and USB cables. The prices of hand-held recording units can widely vary as well, with less expensive units costing less than a hundred dollars and more expensive units exceeding thousands of dollars. Another example worth noting is that we have collected high-quality audio-recordings with inexpensive key chain recorders for pharmacy secret shopper data collection, [33]. The recordings from these keychains were then uploaded to Box, a private data storage server following university-approved HIPPA procedures [33].

### 2.4. Desktop Capture Software

We found that desktop capture software proved useful in facilitating PPBR data collection and training research personnel [16,23]. Potentially available through researchers’ organizations/universities, desktop capture software can be still (i.e., capture of a single screenshot image), or dynamic (i.e., recording screen movement over a period of time). Still desktop capture software, like HyperSnap, enables the capture and editing of high-quality Windows screenshots. Resembling Windows Paint, this software is easy to use and requires little training. HyperSnap is specifically useful to PPBR, because of its ability to protect protected health information (PHI) as once black boxes are placed and saved on screenshots, it is unable to be moved or deleted. This function can help prevent the accidental disclosure of HIPAA-protected information through unintentional editing of the file. As an example of HyperSnap’s use in PPBR, HyperSnap was used by the authors to study how clinical decision support (i.e., alerts/flags) for medication therapy management (MTM) was designed and delivered in community pharmacy practice. We researchers trained pharmacists to capture screenshots of MTM alerts using HyperSnap as they worked up patients in their everyday practice. Pharmacists were able to redact patient identifiers to ensure patient confidentiality, and then submit screen shots to us investigators for analysis. The results of this research were applied to develop recommendations for MTM clinical decision-support redesign to aid more effective care delivery [16,23].

Contrary to still desktop capture software, we found that dynamic desktop capture programs, like MediaSite, Morae and Kaltura, allow researchers to record screen movements. Because users can record audio along with screen movement, organizations/universities often make these programs available to academics for recording lectures and class sessions. However, in addition to being useful as a teaching aid, we found that dynamic desktop capture software can also prove useful in developing training videos for research personnel, especially for training in other technologies described in this paper, like e-forms/surveys software [31]. However, we experienced that the drawbacks of using dynamic desktop capture software to create training materials mirrored the same drawbacks of using video recording software, including editing challenges [31].

Furthermore, we found that dynamic desktop capture software can be useful in facilitating usability studies. For example, when used in conjunction with web-conferencing software (e.g., WebEx, as described below), pharmacist-study participants in our MTM alert study were able to share their real-time, computer work with researchers, who in turn were able to record the user’s on-screen interaction via Morae [16]. This technology was also used in combination with video/audio recording software (i.e., computer-based webcams) to allow us researchers to record participants’ faces and reactions to using software as they “thought aloud.” As such, this is just one example where several different technologies can be used synergistically.

### 2.5. Web-Conferencing and Group-Messaging Applications

We found that electronic-communication technologies, like web-conferencing applications (e.g., Skype, WebEx, Zoom, FaceTime) and group-messaging applications (e.g., WhatsAPP, GroupMe) can be helpful in maintaining coordination among remote study sites [31,32]. Keeping all the key and non-key personnel abreast of study-related activities is a common challenge to PBRN research as study sites and team members are physically distant from each other; e-communication applications directly address this challenge and offer several benefits over traditional communication techniques. In addition to features like complete end-to-end security, unlimited sessions/messages, and e-invites/reminders, we found that e-communication applications offer researchers benefits because they are accessible via a variety of sources like desktops, smartphones, tablets and other mobile devices. Moreover, we found that e-communication applications are often free, user friendly, require minimal set up and can be used by many participants simultaneously [31].

We found that web-conferencing applications’ most notable benefit beyond typical conference calls is their ability to facilitate face-to-face communication; this is particularly important as face-to-face communication is known to increase team-member satisfaction and a sense of unity beyond non-face-to-face communication. Applications like Skype, WebEx and FaceTime can also be used to facilitate semi-structured interviews and focus groups because unlike typical conference calls, web-conferencing applications often allow users to share their on-screen view, record calls and exchange documents. Similarly, compared to typical emails, we found that group-messaging applications’ most notable benefit is their ability to spread information quickly and in real-time among multiple people, without multiple long email-chains. Group-messaging applications can also show read receipts with time-stamps, and may provide faster, more concise responses than email [31].

Despite providing researchers with multiple benefits, web-conferencing and group-messaging applications are not without their drawbacks. Firstly, the applications require devices with internet connection, and conferencing sessions require devices with audio and video functions. Similarly, while applications rarely limit users on the number of conferences/messages sent, some free versions may limit the time conferences may last or the number of participants. Furthermore, we found that if research personnel communicate via group-messaging applications, but are not issued work-related cellphones, personal devices must be used; multiple group-messaging replies—like email—can become a nuisance. More concerning, however, web-conferencing and group-messaging applications present privacy concerns, as users must accept the Terms of Use without HIPAA-compliant levels of privacy guaranteed.

### 2.6. Data Storage and File Transfer Programs

Typically, organizations/universities provide researchers with secure, password-protected servers, platforms and encrypted email programs that allow them to store data securely and share/collaborate on files with individuals from their own institution (e.g., Microsoft Office applications). However, we encountered challenges when attempting to transfer, share or collaborate on files with other individuals outside of our universities or use platform-based technology that remotely stores data (e.g., InfraWare). Specifically, we recognized that emails cannot transfer large files, and flash drives risk data losses and breaches of confidentiality. Similarly, while publicly available web-based file storage and sharing platforms like Google Drive and Dropbox are available to researchers for free, we found that they may offer little security unless individually contracted with our respective university. To address these challenges, we authors used file transfer programs (FTPs) which are web-based programs that provide short-term storage and encryption specifically for secure file transfer.

One example of an FTP we used is FileLocker. FileLocker was developed at Purdue University but is available among multiple universities worldwide [34]. The application provides encryption and is compliant for sharing HIPAA- and FERPA-protected files alike. Researchers may use FileLocker to request files from public users and create distribution accounts that other users can access; these features are particularly helpful when transferring protected data among institutions, or to approve outside third parties. For example, in our previous research we used FileLocker to exchange identifiable audio-recordings and transcripts between universities and private transcription service companies [35]. FileLocker also provides an extra level of security above and beyond traditional data-sharing methods as it allows researchers to audit user activity.

## 3. Challenges of Using Technology in Pharmacy Practice-Based Research

Despite technology’s ability to facilitate PPBR, we experienced unintended consequences, challenges and limitations to its implementation (Table 2). We found that these challenges require investigators to work closely with their institutions’ regulatory and/or information technology teams to ensure the successful use of technology in PPBR. Firstly, the use of technology raises concerns about privacy, especially that of protected health information (PHI). We urge other researchers to not only read and understand a vendor’s privacy statements, but also be aware that vendors reserve the right to revise these agreements upon notice. Furthermore, no technology is immune from unwanted interception by third parties. As such, we suggest that researchers using any web or electronic platform to collect, store, transmit or otherwise handle data should be careful not to make guarantees of confidentiality or anonymity. Rather, researchers should be transparent during informed consent about the limits of confidentiality. Similarly, we suggest that researchers can protect privacy by either removing identifiable data entirely or otherwise separating identifiers from data and linking the two with unique study identifiers and/or code-keys. Code-keys should be stored independently and separately from any corresponding identifiable information and study data.

In addition to privacy concerns, researchers may face challenges understanding, applying for—and complying with—institutional review boards (IRBs) when attempting to use technology in their studies. Studies using technology for human subject research must be approved by IRBs, be HIPAA-compliant and comply with all the U.S. Department of Health and Human Services’s Protection of Human Subjects federal regulations, just like any other research. Therefore, researchers are responsible for explaining to their IRBs how the proposed technology provides the same level of protection as traditional methods. Researchers should be able to easily find these explanations in plain language in technology vendors’ Terms of Use; if, however, such language is neither present nor readily understandable in the Terms of Use, researchers may take heed of using that technology in favor of another option.

The integration of technology in PPBR is also challenging because electronic data files can become corrupt. Years of researchers’ work can be immediately invalidated by malware, viruses, physical damage or by the simple aging of obsolete software. Saving multiple copies of files on independent platforms, servers and drives reduces the risk of data loss, but this practice inversely increases the risk of a loss of privacy through data breach. Conversely, researchers may be wary of using technology in research because electronic deletion is neither readily visible nor verifiable. Furthermore, once researchers utilize a certain technology, they must then rely on an uncontrollable entity (i.e., technology vendors) to continue to support and maintain that product. In other words, most technology vendors are private businesses, and therefore reserve the right to change, or discontinue any service or product they produce. Naturally, researchers can be uncomfortable with this dependence. Indeed, PPBR activities are limited by a lack of adequate resources, and researchers with low-technology skills (either actual or perceived) may then further shy away from using technology as little IT resources may be available to offer support.

Technology’s usefulness in facilitating PPBR is also limited because sometimes the technology itself is simply not well advanced. For example, the InfraWare Dictation recording and transcription application utilizes professional human transcriptionists to transcribe audio recordings to text. While artificial intelligence dictation tools exist, we authors found in our research that these technologies are not advanced enough to reliably and correctly transcribe audio-recordings. Naturally, voice recognition software is expected to advance to a point where it will be regularly helpful in PPBR because most technology tends to advance at an exponential rate. However—regardless of technology’s future advancement and ability to facilitate PPBR—its use will likely remain challenging because advancement and cost go hand in hand. As such, there is a lag period between when a new technology is developed and when it becomes readily accessible.

An example of this lag period’s negative effect on PPBR is represented by internet accessibility; the lack of wireless internet access is a known challenge to conducting PPBR. Wireless internet was first demonstrated in 1971, but many community pharmacies have neither internet nor Wi-Fi, choosing rather to rely on intranets (i.e., networking and sharing systems that resemble the internet, but are bound to a single entity). Costs and dissemination/implementation challenges are not the only reason why community pharmacies often utilize intranet over internet. Pharmacies may restrict internet access to employees for security or productivity reasons. Consequently, some PPBR challenges related to human factors may continue to exist regardless of technology’s advancement, costs and accessibility.

## 4. Discussion

Here, we authors presented our experiences and lessons learned in applying technology-driven solutions to overcome common PPBR challenges. From these experiences, we believe that the benefits and unintended drawbacks of technology’s integration into health systems may oscillate, but that ultimately technology’s role in healthcare will only continue to become more vital. Indeed, American pharmacies universally rely on computerized systems to dispense medications and deliver innovative services. As such, we hope to share these experiences so that other pharmacy academics can capitalize on technologies to overcome common practice-based research barriers, such as research team coordination, unique data collection needs and privacy concerns.

Given enough time, it seems plausible that technology could eventually address most PPBR barriers because technology ‘grows’ at an exponential rate. For example, the old adage ‘Moore’s Law’ states that circuit transistors double every two years. Furthermore, promising tools like machine learning and artificial intelligence have the potential to greatly improve future academics’ utilization and understanding of PPBR. However, technology is not likely to become the ultimate liberator for pharmacy practice-based researchers’ challenges (at least in this era) because many challenges either relate to or stem directly from social, economic or human factors. For example, one of the most visible and well known barriers to PPBR is the need to minimize workflow disruptions as sites continue to deliver their regular services [36]. PPBR research sites primarily exist to deliver health services, and are not sterile, controllable environments that are readily primed to carry out research; so, while technology-driven solutions can reduce workflow disruptions caused by traditional PPBR methods, we believe that these solutions often create other workflow concerns. Respect for our pharmacy colleagues’ work realities and priorities will continue to be at the center of our successful PPBR collaboration.

We believe that another PPBR barrier unlikely to be overcome with technology in the near future is the discipline’s inability to perform meaningful systematic reviews and meta-analysis on pharmacist-provided services. Ideally, advancements in artificial intelligence (AI) are predicted to assist researchers perform meta-analysis/systematic review by identifying suitable articles, collecting/analyzing data and examining sources of heterogeneity between studies. However, it is likely that if (or when) any such AI is developed, PPBR academics will continue to face challenges completing systematic reviews and met-analysis of pharmacy practice services because of the misuse, confusion and variable use of pharmacy practice service terminology in the literature. Little standardized MeSH (Medical Subject Headings) terms exist that readily encompass modern pharmacy practice, especially regarding community-based services, and the profession has adopted no universal definitions for its services [37]. Accordingly, PPBR researchers will likely need to wait until the AI is advanced enough to understand subtitles in human language patterns and analogies for assistance in systematic reviews and meta-analysis [38].

Indeed, as this paper attempted to outline, our experiences have shown that technology’s use in facilitating PPBR appears as a double edged sword (Table 1); whereas technology may assist in overcoming a barrier, a new concern is raised. As such, while technology can facilitate the speed of data collection and transfer, privacy concerns are raised. Similarly, while researchers have more technological solutions to choose from than ever before (and new products being developed rapidly), laws, regulations and researchers’ comfort using such technology may not keep the pace. Ultimately, it is almost certain that clinicians and researchers will continue to use technology, and that this use not only provides unique research opportunities for academics, but also to propel the profession of pharmacy forward.

## 5. Limitations

This paper is limited because the experiences presented are merely our own and do not necessarily represent others’ experiences with the same or similar technology. As such, all authors’ experiences took place within the United Stated circa 2010–2020, so the lessons learned in implementing these technology-driven solutions are less applicable to researchers outside of the United States. Similarly, this paper’s applicability will most likely diminish as time progresses, as newer versions of technology and strategies to implement those technologies are made. Furthermore—as the experiences presented are only our own—we make no guarantee, endorsement of, nor discourage the use of any singular product.

Furthermore, we authors do not claim to be pioneers in the use of these technologies in overcoming PPBR challenges. Conversely, the tools we reported on here were brought to our attention initially because they held pre-existing reputations among research communities, and we emphasize that we simply used specific products as they were available at our universities; comparable products available elsewhere could likely be explored and used too. Therefore, many specific technologies, products or tools were never mentioned or considered as they were not used by the authors.

## Figures and Tables

**Table 1:** Authors’ experiences with technology organized by the Technology Acceptance Model’s “perceived usefulness” and “perceived ease of use” constructs with supporting examples.

Common PPBR * Barrier	Authors’ Perceived Usefulness of Technology	Authors’ Perceived Ease of Use of Technology to Overcome Barrier		Examples	Cited Work
		Positive	Negative		
Physical Distance from Research Team	Group messaging and e-conference software can provide needed information “just in time,” facilitate positive team dynamics with face-to-face interaction and avoid unnecessary emails.	Group-messaging applications are no more difficult to use than regular mobile-plan or SMS text messaging. E-conference software is usually user-friendly and uses prompts to facilitate meetings. Recording data for study purposes requires no different use than for e-conferencing.	Ease of use steadily decreases with any single research team-member’s inability to use the technology (e.g., muting lines on shared conferences, failing to enable notifications). Privacy concerns may limit use.	**WebEx:** webex.com **WhatsAPP:** whatsapp.com **Zoom:** zoom.us	[16,31,32]
Lack of Internet Access in Community Pharmacies	Researchers can work around a lack of internet access by enabling software programs’ “offline modes” and/or deploying their own hot-spot devices.	Turning on personal hotspot data or enabling ‘offline-modes’ can be as simple as pushing a single button.	Cost of hotspot devices and accompanying data plans may be cost prohibitive. Users must link their devices to the hotspot connection. Researchers must remember to manually download data collected in programs’ ‘offline modes” and extract it to data repositories.	REDCap: project-redcap.org Mobile Hotspot (Sprint): sprint.com	[15,16]
Lack of Trained, On-Site Research Personnel	Smartphones, tablets and other mobile devices, with or without the use of desktop capture software, can be used to make training videos. Videos last as enduring education and can be particularly useful with process/spatial training.	Videos are easy for trainees to use, and can be exceptionally useful for rotating, temporary or remotely-located research personnel. Academics often have experience using desktop capture and audio recording software for teaching purposes.	Some knowledge of video-editing software is needed, and editing videos can be time consuming and difficult. Required hardware can be cost prohibitive.	GoPro: gopro.com Kaltura: kaltura.com WebEx: webex.com	[16,31,32]
Recruiting, and Consenting Study Participants (Including Non-English Speakers)	Electronic data collection form/survey software can be used for screen, recruit and consent purposes. On-site research personnel can use handheld devices to access secure sharing platforms or e-forms/surveys containing telephone contact with off-site translators, and brief translated recruitment materials.	Electronic informed consent documents are saved in the same manner as any other document in these programs. Pre-translated consent materials are readily retrievable. Off-site translators are able to readily access all consent materials through a mutual Box Drive.	Some platforms can only be accessed via a desktop, limiting their use. Non-English speakers can be hesitant about consenting to use of technology when approached by a sole English speaker due to sociocultural differences.	REDCap: project-redcap.org Box: box.com	[15,16,24]
Primary Data Collection	E-form/survey software, with or without the use of mobile devices can aid in remote data collection and transfer.	Use of institutions’ “preferred” software programs allows researchers easy access and security assurance. Creation and use of e-forms/surveys are fairly intuitive. Programs provide many easy-to-use, customizable features. Data is downloadable in various formats and some descriptive statistics may be auto-generated.	IT help may not always be available if needed, and professional IT help may be needed to move e-forms/surveys from ‘development’ to ‘production.’ Fees can be applied per survey and e-forms/surveys can expire. Electronic systems like mobile devices and computers are needed to access the e-form/survey software. Researches may need to maintain a code-keys to link identifiable and de-identifiable data.	Qualtrics: qualtrics.com REDCap: project-redcap.org Tablets (Apple): apple.com Tablets (Samsung): samsung.com	[4,11,15,16,17,18,19,20,21,22,23,27]
Unique Data Collection Needs	Combinations of technologies can be used in synch to collect unusual data. Researchers can avoid using handheld audio-recorders by using mobile-applications.	Tablet-based technology easily collect patient-reported outcomes in tech-savvy populations. Mobile applications have fairly user-friendly interfaces and real-time progress on transcription can be seen.	Mobile applications may not perform optimally when other applications are running. Human transcription services are still needed as auto-transcription software is unreliable.	WebEx webex.com InfraWare: infraware.com	[15,23,28,29,30,33]
Data Security and Transfer	Researchers can avoid loss of data and data breaches by transferring files with file transferring programs (FTPs).	FTPs are usually as easy to use as any other cloud-based file storage platform.	Users need to register accounts. Files are automatically deleted for safety purposes after a set amount of time.	FileLocker: filelocker2.sourceforge.net	[35]

* Pharmacy Practice-Based Research (PPBR).

**Table 2:** Authors’ experiences overcoming the challenges of implementing technology-driven solutions in pharmacy practice-based research.

Major Lesson	Experiences Overcoming Implementation Barriers
Contact Institutional Review Boards and IT Departments Before Writing Protocols. Utilize Academic Institution’s Preferred Software Programs.	Academic institutions usually have preferred software programs and these are often available to PPBR researchers for little to no-charge and may offer extra features, security, and/or support. Avoid publicly available software programs, as paywall and privacy issues may arise. Academics may save effort obtaining IRB approval if utilizing their institution’s preferred software, as IRB members will likely be familiar with such preferred programs. Software programs may assert HIPAA compliancy; however, researchers may save time and undue delays by contacting IRBs before developing protocols. Development and maintenance of positive working relationships with IT departments is crucial; IT personnel should be consulted before using any new software and notified of any changes.
Prepare for Technologies’ Limitations. Take Redundant Efforts to Protect e-Data.	Develop back-up protocols with traditional resources. Avoid breaches and data loss file-monitoring and protection software and regularly deploy antivirus and malware programs. Maintain the latest versions of vendor-supplied security patches. Read technology vendors’ Terms of Use and/or Privacy Policies before choosing products and cite these documents in IRB applications. Be aware of any clauses that allow vendors to change Terms and Policies, and how they may be changed.

## References

[1] United States Agency for Healthcare Research and Quality Practice Based Research Networks. https://pbrn.ahrq.gov/.

[2] United States Centers for Disease Control and Prevention (2014). How Pharmacists Can Improve Our Nation’s Health. https://www.cdc.gov/grand-rounds/pp/2014/20141021-pharmacist-role.html.

[3] Patel P., Hemmeger H., Kozak M.A., Gernant S.A., Snyder M.E. (2015). Community pharmacist participation in a practice-based research network: A report from the Medication Safety Research Network of Indiana (Rx-SafeNet). J. Am. Pharm. Assoc..

[4] Kozak M.A., Gernant S.A., Hemmeger H.M., Snyder M.E. (2015). Lessons learned in the growth and maturation stages of a community pharmacy practice-based research network: Experiences of the Medication Safety Research Network of Indiana (Rx-SafeNet). Innov Pharm..

[5] Davis F.D. (1989). Perceived usefulness, perceived ease of use, and user acceptance of information technology. MIS Q..

[6] Taherdoost H. (2018). A review of technology acceptance and adoption models and theories. Procedia Manuf..

[7] Strudwick G. (2015). Predicting nurses’ use of healthcare technology using the technology acceptance model: An integrative review. Comput. Inform. Nurs..

[8] Ketikidis P., Dimitrovski T., Lazuras L., Bath P.A. (2012). Acceptance of health information technology in health professionals: An application of the revised technology acceptance model. Health Inform. J..

[9] Pai F.-Y., Huang K.-I. (2011). Applying the technology acceptance model to the introduction of healthcare information systems. Technol. Forecast Soc. Chang..

[10] Riad M., Jaradat M.-I., Moh Z., Ziad S. (2013). Applying the technology acceptance model to the introduction of mobile healthcare information systems. Int. J. Behav. Healthc. Res..

[11] Chen A.M., Kiersma M.E., Shepler B.M., Murawski M.M. (2013). Pilot testing of checklists to discern adverse drug reactions and adverse drug events. J. Am. Pharm. Assoc..

[12] Clayman M.L., Bylund C.L., Chewning B., Makoul G. (2016). The impact of patient participation in health decisions within medical encounters: A systematic review. Med. Decis. Mak..

[13] Schommer J.C., Olson A.W., Isetts B.J. (2019). Transforming community-based pharmacy practice through financially sustainable centers for health and personal care. J. Am. Pharm. Assoc..

[14] Schommer J.C., Doucette W.R., Planas L.G. (2015). Establishing pathways for access to pharmacist-provided patient care. J. Am. Pharm. Assoc..

[15] Snyder M.E., Chewning B., Kreling D., Perkins S.M., Knox L.M., Adeoye-Olatunde O.A., Jaynes H.A., Schommer J.C., Murawski M.M., Sangasubana N. (2020). An evaluation of the spread and scale of PatientToc™ from primary care to community pharmacy practice for the collection of patient-reported outcomes: A study protocol. Res. Soc. Adm. Pharm..

[16] Snyder M.E., Adeoye-Olatunde O.A., Gernant S.A., Dilulio J., Jaynes H.A., Doucette W.R., Russ-Jara A.L. (2020). A user-centered evaluation of medication therapy management alerts for community pharmacists: Recommendations to improve usability and usefulness. Res. Soc. Adm. Pharm..

[17] Hale J.C., Murawski M.M., Ives T.J. (2015). Practice characteristics and geographic distribution of clinical pharmacist practitioners in North Carolina. N. C. Med. J..

[18] Hale J.C., Murawski M.M., Ives T.J. (2013). Perceived successes and challenges of clinical pharmacist practitioners in North Carolina. J. Am. Pharm. Assoc..

[19] Gernant S.A., Zillich A.J., Snyder M.E. (2018). Access to medical records’ impact on community pharmacist-delivered medication therapy management: A pilot from the Medication Safety Research Network of Indiana (Rx-SafeNet). J. Pharm. Pract..

[20] Snyder M.E., Jaynes H.A., Gernant S.A., Lantaff W.M., Doucette W.R., Hudmon K.S., Perkins S.M. (2020). Factors associated with comprehensive medication review completion rates: A national survey of community pharmacists. Res. Soc. Adm. Pharm..

[21] Wellman B.R., Frail C.K., Zillich A.J., Snyder M.E. (2015). Pharmacists’ experiences with a telephonic medication therapy management program for home health care patients. Consult. Pharm..

[22] Noureldin M., Murawski M.M., Mason H.L., Plake K.S. (2013). Student pharmacists’ attitudes toward complementary and alternative medicine. J. Am. Pharm. Assoc..

[23] Snyder M.E., Jaynes H., Gernant S.A., DiIulio J., Militello L.G., Doucette W.R., Adeoye O.A., Russ A.L. (2019). Alerts for community pharmacist-provided medication therapy management: Recommendations from a heuristic evaluation. BMC Med. Inform. Decis. Mak..

[24] Harris P.A., Taylor R., Thielke R., Payne J., Gonzalez N., Conde J.G. (2009). Research electronic data capture (REDCap)—A metadata-driven methodology and workflow process for providing translational research informatics support. J Biomed Inform..

[25] McCall B. (2018). What does the GDPR mean for the medical community?. Lancet.

[26] Tanavalee C., Luksanapruksa P., Singhatanadgige W. (2016). Limitations of using Microsoft Excel version 2016 (MS Excel 2016) for statistical analysis for medical research. Clin. Spine Surg..

[27] Kozak M.A., Melton J.R., Gernant S.A., Snyder M.E. (2016). A needs assessment of unused and expired medication disposal practices: A study from the Medication Safety Research Network of Indiana. Res. Soc. Adm. Pharm..

[28] Shah B., Chewning B. (2006). Conceptualizing and measuring pharmacist-patient communication: A review of published studies. Res. Soc. Adm. Pharm..

[29] Lor M., Chewning B. (2016). Telephone interpreter discrepancies: Videotapes of Hmong medication consultations. Int. J. Pharm. Pract..

[30] McPherson K.L., Adeoye-Olatunde O.A., Osborne J.M., Doucette W.R., Gernant S.A., Jaynes H., Phansalkar S., Russ-Jara A.L., Snyder M.E. (2020). Community pharmacists’ and residents’ decision making and unmet information needs when completing comprehensive medication reviews. J. Am. Pharm. Assoc..

[31] Perry S.E., Fragomeli M., Kurzatkowski A., Gernant S.A. (2018). Integrating Meds to Beds Services from a Community Pharmacy during Inpatient Discharge (Poster).

[32] Choi A., Mierz S., Gernant S.A. (2019). Students’ Perceptions of Barriers and Facilitators in Leading a Community-Pharmacy Transitions-of-Care Service (Poster).

[33] Peters J., Desai K., Ricci D., Chen D., Singh M., Chewning B. (2016). The power of the patient questions: A secret shopper study. Patient Educ. Couns..

[34] Brett D., Filelocker C.M. 2006. http://filelocker2.sourceforge.net/.

[35] Snyder M.E., Jaynes H.A., Gernant S.A., Lantaff W.M., Hudmon K.S., Doucette W.R. (2018). Variation in Medication Therapy Management Delivery: Implications for Health Care Policy. J. Manag. Care Spec. Pharm..

[36] Reddy A., Abebe E., Rivera A.J., Stone J.A., Chui M.A. (2019). Interruptions in community pharmacies: Frequency, sources, and mitigation strategies. Res. Soc. Adm. Pharm..

[37] Gernant S.A., Bacci J.L., Upton C., Ferreri S.P., McGrath S., Chui M.A., Rickles N.M., Smith M. (2019). Three opportunities for standardization: A literature review of the variation among pharmacists’ patient care services terminology. Res. Soc. Adm. Pharm..

[38] Thomas J., Noel-Storr A., Marshall I., Wallace B., McDonald S., Mavergames C., Glasziou P., Shemilt I., Synnot A., Turner T. (2017). Living Systematic Review Network. Living systematic reviews. Combining human and machine effort. J. Clin. Epidemiol..

